# Extracranial-intracranial bypass surgery for intracranial aneurysm of the anterior cerebral circulation: A systematic review and meta-analysis

**DOI:** 10.3389/fneur.2023.1174088

**Published:** 2023-03-31

**Authors:** Yang Chen, Pengyu Chen, Guosheng Duan, Ren Li, Ziao Li, Geng Guo

**Affiliations:** ^1^Department of Neurosurgery, The First Hospital of Shanxi Medical University, Taiyuan, Shanxi, China; ^2^Shanxi Provincial People's Hospital, Shanxi Medical University, Taiyuan, China; ^3^Department of Emergency, The First Hospital of Shanxi Medical University, Taiyuan, Shanxi, China

**Keywords:** intracranial aneurysm, extracranial-intracranial bypass, cerebral revascularization, anterior cerebral circulation, management

## Abstract

**Background:**

The safety of extracranial–intracranial (EC–IC) bypass in the management of anterior circulation intracranial aneurysms (IAs) remains to be determined. This systematic review aims to summarize the existing evidence and provide guidance for the precise management of IAs.

**Data source:**

We constructed search strategies and comprehensively searched Pubmed, Medline, Embase, Web of science, and Cochrane library.

**Methods:**

This systematic review was actualized according to the PRISMA statement. We evaluated study quality using the methodological index for non-randomized study (MINORS). Effect sizes were pooled using a random-effects model. Heterogeneity between studies was assessed using the *I*^2^ test. Publication bias was assessed using the Egger's test. The registration number for this systematic review is CRD42023396730.

**Result:**

This systematic review included a total of 21 articles, involving 915 patients. Postoperative bypass patency rate was 99% (95% CI 0.98–1.00); short-term follow-up was 98% (95% CI 0.94–1.00); long-term follow-up was 95% (95% CI 0.93–0.97). The long-term follow-up occlusion rate of saphenous vein was higher than that of radial artery (OR 6.10 95% CI 1.04–35.59). Short-term surgery-related mortality was 0.3% (95% CI 0.000–0.012); long-term follow-up was 0.4% (95% CI 0.000–0.013); The proportion of patients with a score of 0–2 on the modified Rankin Scale (mRS) during long-term follow-up was 92% (95% CI 0.86–0.98). The incidence rates of long-term follow-up complications were: ischemic 3% (95% CI 0.01–0.06); hemorrhagic 1% (95% CI 0.00–0.03); neurological deficit 1% (95% CI 0.00–0.03); other 3% (95% CI 0.01–0.06).

**Limitation:**

Most of the included studies were retrospective studies. Studies reporting preoperative status were not sufficient to demonstrate postoperative improvement. Lack of sufficient subgroup information such as aneurysm rupture status.

**Conclusion:**

EC–IC therapy for anterior circulation IAs has a high safety profile. Higher level of evidence is still needed to support clinical decision.

**Systematic review registration:**

https://www.crd.york.ac.uk/prospero/display_record.php?ID=CRD42023396730, identifier: CRD42023396730.

## 1. Introduction

Regarding the optimal management of intracranial aneurysms (IA), evidence such as large randomized controlled trials is still lacking, thus controversy continues. Because most of the IAs are not symptomatic, the managements of unruptured IA are mostly prophylactic to avoid subarachnoid hemorrhage after IA rupture. However, preventive management does not always benefit patients, and some patients have significantly reduced life satisfaction (1). It is indispensable to consider the patient's wishes and make optimal management individually. The results of the International Subarachnoid Aneurysm Trial (ISAT) have made endovascular therapy the most popular management for IA, especially for small saccular aneurysms of the anterior circulation (2). Endovascular therapy is non-inferior to craniotomy but less invasive. The subsequent emergence of flow diverter (FD) such as Pipeline™ embolization device and Tubridge™ has brought new options for the management of wide-necked giant IA (3, 4). Nonetheless, treatment for giant and complex IAs is still a thorny issue. The presence of perforating arteries in the dome and neck of the giant saccular aneurysm and the irregular shape of the fusiform aneurysm result in persistent aneurysm filling after stenting and limited therapeutic benefit (5). Even Pipeline™ embolization devices weren't perfect for fusiform aneurysms treatment (6). Due to the compression symptoms caused by its mass effect or risk of complications such as thrombus and dissection, surgical relief is required, and mere endovascular treatment is no longer applicable (7). However, microsurgical clipping is unpractical to completely remodel the lumen of a dilated artery and ensure the patency of the parent artery, especially when the IA surrounds a branch artery (8). In addition, calcification and atherosclerosis of the arterial wall and intraluminal thrombosis in complex IAs increase the risk of microsurgical clipping and endovascular therapy (9). In these conditions, occlusion of the parent artery is the last option to completely isolate the aneurysm from the circulation and prevent hemorrhage. Adjunctive bypass surgery can supply the distal branch feeding areas without adequate collateral flow (10).

Since Yasargil described the first case of extracranial–intracranial (EC–IC) bypass surgery for the treatment of IA in 1969, the role of bypass surgery as an adjuvant therapy to ensure the cerebral blood supply is still irreplaceable (11, 12). Controversy persists over the choice of bypass type. In clinical practice, physicians seem to prefer intracranial–intracranial (IC–IC) bypass surgery because it is associated with higher bypass patency rates and lower complication rates (13). Compared with EC–IC bypass, IC–IC bypass has the inherent advantages of needless to harvest and process donor vessels, shorter graft, and less susceptible to neck torsion, injury, and compression obstruction (13). However, EC–IC is irreplaceable in the treatment of IAs proximal to the internal carotid bifurcation. Moreover, in view of the operating depth of IC–IC bypass and the limited range of intracranial arteries movement, EC–IC bypass is easier to master and a safer technique for most doctors (14). In recent years, more literature reports are focused on EC–IC bypass surgery, suggesting uncertainty on its safety (15).

To clarify the safety of EC–IC bypass in the management of IA of the anterior circulation, we conducted this systematic review to synthesize existing evidence and provide guidance for optimal management.

## 2. Materials and methods

### 2.1. Search strategy

This systematic review conducted according to the PRISMA statement (16). The review protocol was registered in the PROSPERO database and is available online (CRD42023396730; https://www.crd.york.ac.uk/prospero/display_record.php?ID=CRD42023396730). The databases Pubmed, Medline, Embase, Web of science, and Cochrane library were systematically searched for all study published from 1980 to December 2022 that evaluated outcomes of EC–IC bypass therapy for anterior cerebral circulation IAs. Keywords for constructing search strategies include “intracranial aneurysm,” “anterior cerebral circulation,” and “cerebral revascularization.” Full search queries are provided in the Supplementary material. We also checked studies in systematic reviews and literature reviews for potential sources.

### 2.2. Outcome definitions

Primary outcomes of the study included bypass patency rate, procedure-related mortality, and neurological function scale scores such as Glasgow Outcome Scale (GOS) and Modified Rankin Scale (mRS) at any follow-up period. Secondary outcomes were defined as the incidence of various surgical-related complications. Complications were divided into four categories including ischemic, hemorrhagic, neurological deficit and others (Deep vein thrombosis and infection et al.). Short-term follow-up is defined as within 30 days, and long-term follow-up is more than 12 months.

### 2.3. Inclusion and exclusion criteria

Studies included in this review had to meet the following criteria: (1) studies reported at least one primary outcome of EC–IC bypass surgery for anterior cerebral circulation IAs; (2) any type of study is qualified (prospective or retrospective); (3) if a study included aneurysms located outside the anterior cerebral circulation, or included other treatment groups, the original text should describe the results of EC–IC bypass for IAs in the anterior cerebral circulation group separately; (4) the target cohort should be not <20 patients. Studies will be excluded if they meet the following criteria: (1) type of publication is review, letter, meta-analysis, case report or comment; (2) non-English publications; (3) patients under the age of 18; (4) abstract only, original text not available; (5) The bypass technique is non-conventional, such as excimer laser-assisted non-occlusive anastomosis. (5) Studies reporting results from overlapping patient cohorts. Patients from different studies were considered overlapping patient cohorts if they were drawn from the same institution or database for the same time period.

### 2.4. Data extraction

Four authors (Y.C., P.Y.C., G.S.D., and G.G.) independently performed literature search and study selection. Disagreements were resolved by consensus and consultation with senior investigators. The text, tables, images, and Supplementary material of the literature were checked to ensure data integrity. Extracted data includes publication information (first author, published year, country, journal, and design type), basic demographics (number of patients, number of procedures, age, gender, aneurysm location, aneurysm size, and follow-up time), bypass type (high flow, low flow and type of graft), bypass patency rate, mortality rate, GOS or mRS score, complication rate. Since the included studies used different internal carotid artery (ICA) segmentation methods, we classified all the proximal ICA bifurcation and its branches as ICA. Most studies considered the posterior communicating artery (PcoA) as part of the anterior circulation, so we included PcoA aneurysms (17, 18). The anterior communicating artery was classified as the anterior cerebral artery (ICA). We divided postoperative complications into four categories. The ischemic complications include transient ischemic attack (TIA), vasospasm, cerebral infarction, low flow–related ischemic complications (LRICs), etc. Hemorrhagic complications include subarachnoid hemorrhage (SAH), intracranial hematoma, aneurysm rupture, etc. Neurological deficits include cranial nerve palsy, disturbance of consciousness, hemiplegia, etc. Other complications include deep vein thrombosis (DVT), infection, wound dehiscence, CSF leak, etc. If there was sample overlap between multiple studies, we included the first published study or the only study for which the primary outcome was available.

### 2.5. Quality assessment

Three authors (Y.C., P.Y.C., and G.S.D.) independently assessed study quality and differences were resolved by consensus. Studies were assessed according to the methodological index for non-randomized study (MINORS) scale. MINORS is a scale for evaluating non-randomized controlled studies in surgery (19). The scale contains 8 items evaluating non-comparative studies and 4 additional items evaluating comparative studies. Therefore, the maximum score for the study is 16 or 24 points respectively. Baseline characteristic data expressed as mean ± standard deviation or median (range), event rates converted to number of events (percentage).

### 2.6. Statistical analysis

We aggregated effect size using R software (V.4.2.1) and the R package “meta.” We calculated pooled effect sizes and 95% confidence intervals (CI) for each outcome. Given that most of the studies we included were non-comparative and potential heterogeneity may exist, we used the random-effects model to estimate pooled values. Heterogeneity was assessed using *I*^2^ and 95% CI. Egger's test was used to assess publication bias for pooling ≥5 studies. In addition, the graft occlusion rates of different bypass types were compared using a random-effects model. Both aggregated rates and aggregated odds ratios are presented.

## 3. Result

### 3.1. Characteristics and quality of the included studies

**Figure 1:**
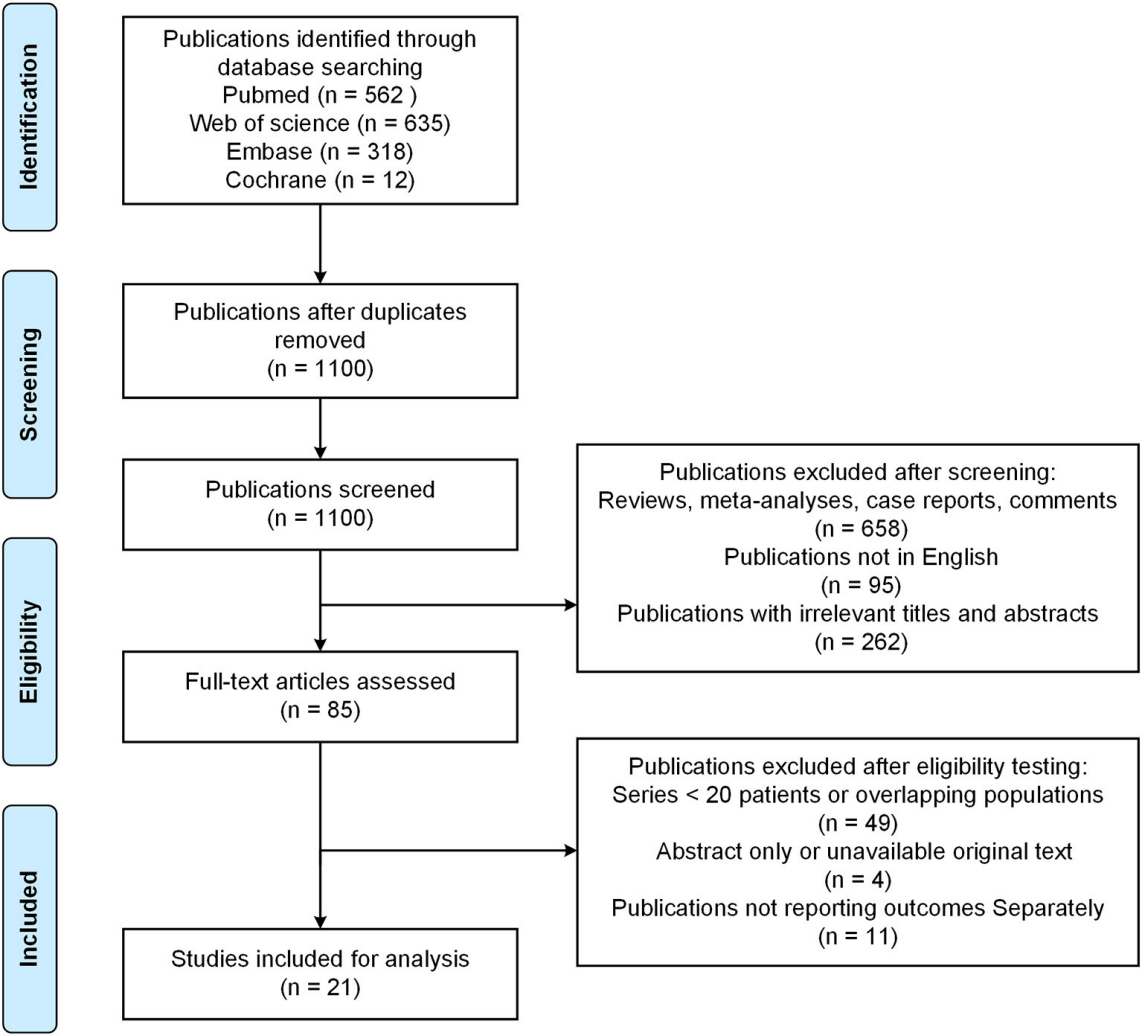
Preferred reporting items for systematic reviews and meta-analysis (PRISMA) flow diagram of this study.

After identification, 21 studies were included in this review, involving a total of 22 cohorts and 915 patients (13, 14, 20–38). The study of Abdulrauf et al. (31) included a prospective cohort of 30 patients and a retrospective cohort of 110 patients. The study by Dodie et al. (38) included one patient with a failed intraoperative bypass, so a total of 40 bypasses were performed. We evaluated a total of 1,100 unique publications and 1,079 were excluded (Figure 1). Studies were published in years ranging from 2002 to 2022. The type of study design included four prospective studies and 17 retrospective studies (Table 1). Nine studies were from the United States, 3 from Japan, 3 from China, 2 from Italy, and the remaining studies were from South Korea, Austria, Australia and Finland (Table 1). Study sample sizes were ranging from 20 to 110. Patient's age and gender information was available for a total of 11 studies. Except for the patients in Natarajan et al. study, the female patients were more than male. The average age of the vast majority of study patients was older than 50 years (Table 1). Aneurysm location information was available for a total of 19 studies, with most of aneurysms located proximal to the ICA bifurcation and its branches. Aneurysm size information was available for a total of 10 cohorts, with the majority of IAs being large (≥10 mm) and giant (≥25 mm) aneurysms. Follow-up duration was described in most studies expect for two studies. A total of 6 studies were comparative and 15 studies were non-comparative. The median score of non-comparative studies was 11 (10–14) and comparative studies was 19.5 (18–22) (Table 1).

**Table 1:** Characteristics and quality of included studies.

**Study**	**Year**	**Country**	**Journal**	**Design**	**Patient/bypass**	**Age**	**Gender (F:M)**	**Aneurysm location**				**Aneurysm size (mm)**	**Follow-up (month)**	**MINORS**
								**ICA**	**MCA**	**ACA**	**PcoA**			
Morgan et al. (20)	2002	AUS	Journal of Clinical Neuroscience	Prospective	21/21	45.61 ± 12.96	12:9	21 (100)	0	0	0	NR	Mean 41.76	14/16
Jafar et al. (21)	2002	USA	Neurosurgery	Retrospective	27/28	NR	NR	20 (71.43)	2 (7.14)	0	6 (21.43)	NR	Mean 62	10/16
Cantore et al. (22)	2008	ITA	Neurosurgery	Retrospective	40/40	NR	NR	40 (100)	0	0	0	NR	Median 102	11/16
Sanai et al. (13)	2009	USA	Neurosurgery	Retrospective	38/38	NR	NR	31 (81.58)	7 (18.42)	0	0	NR	Mean 41	20/24
Murakami et al. (23)	2009	JPN	Surgical Neurology	Retrospective	29/29	57 ± 12.16	28:1	29 (100)	0	0	0	18 (2–58)	NR	11/16
Xu et al. (24)	2011	CHN	The Canadian Journal of Neurological Sciences	Retrospective	22/22	NR	NR	19 (86.36)	3 (13.64)	0	0	NR	Mean 12	10/16
Ramanathan et al. (25)	2012	USA	Neurosurgery	Prospective	30/30	NR	NR	23 (76.67)	7 (23.33)	0	0	NR	Mean 32	22/24
Shi et al. (26)	2014	CHN	Neurosurgical Review	Retrospective	61/61	NR	NR	NR	NR	NR	NR	NR	Mean 36	10/16
Ishishita et al. (27)	2014	JPN	World Neurosurgery	Retrospective	37/37	57.57 ± 11.96	30:7	35 (94.59)	0	0	2 (5.41)	G/L: 25/12	Mean 46.7	18/24
Kalani et al. (28)	2014	USA	Neurosurgery	Retrospective	25/25	50.62 ± 14.87	18:7	17 (68)	7 (28)	1 (4)	0	NR	Mean 18.5	11/16
Rustemi et al. (29)	2015	USA	Neurosurgery	Retrospective	22/22	55.95 ± 18.47	13:9	13 (59.09)	8 (36.36)	0	1 (4.55)	22 (3–40)	Mean 44.8	12/16
White et al. (30)	2016	USA	World Neurosurgery	Retrospective	27/27	50.74 ± 15.61	18:8 NA1	18 (66.67)	8 (29.63)	1 (3.7)	0	26 (8–60)	Minimum 6	11/16
Ban et al. (14)	2017	KOR	Operative Neurosurgery	Retrospective	49/49	NR	NR	35 (71.43)	13 (26.53)	1 (2.04)	0	NR	Mean 34.2	12/16
Abdulrauf et al. (31)	2017	USA	World Neurosurgery	Prospective	30/30	50.1 ± 6.5	17:13	30 (100)	0	0	0	27.9 (20–65)	NR	18/24
				Retrospective	110/110	48.0 ± 7.3	57:53	110 (100)	0	0	0	NR		
Matsukawa et al. (32)	2017	JPN	Journal of Neurosurgery	Retrospective	80/80	59 ± 15	66:14	80 (100)	0	0	0	17 (11–17)	Median 26.1	13/16
Nussbaum et al. (33)	2018	USA	Journal of Neurosurgery	Retrospective	95/95	NR	NR	NR	NR	NR	NR	NR	Minimum 12	19/24
Ni et al. (34)	2018	CHN	World Neurosurgery	Prospective	32/32	NR	NR	0	32 (100)	0	0	NR	Mean 59.4	20/24
Nurminen et al. (35)	2019	FIN	World Neurosurgery	Retrospective	24/28	50.63 ± 16.80	13:11	24 (100)	0	0	0	30 (2–79)	Mean 51	10/16
Natarajan et al. (36)	2019	USA	World Neurosurgery	Retrospective	21/24	50.90 ± 13.93	9:12	0	22 (100)	0	0	12 (3–37)	Mean 39.3	12/16
Pescatori et al. (37)	2021	ITA	World Neurosurgery	Retrospective	55/55	NR	NR	55 (100)	0	0	0	NR	Minimum 12	12/16
Dodier et al. (38)	2022	AUT	Journal of Neurointerventional Surgery	Retrospective	41/40	57 (19–73)	30:11	41 (100)	0	0	0	24 (5–79)	Median 46.8	11/16

Data presented as mean ± standard deviation (SD), median (range), n (%) or otherwise stated. ICA, internal carotid artery; MCA, middle cerebral artery; ACA, anterior cerebral artery; PcoA, posterior communicating artery; G/L, giant/large; NR, non-reported.

### 3.2. Bypass patency and mortality

**Figure 2:**
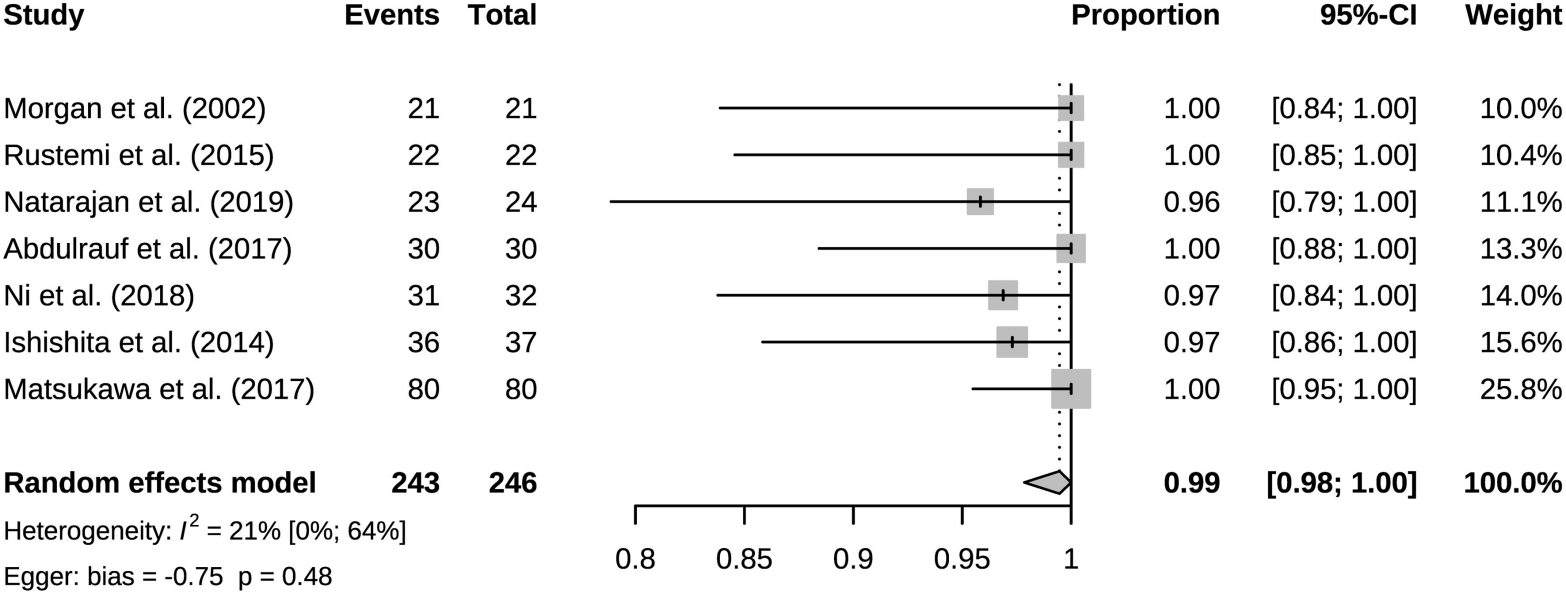
Immediate postoperative bypass patency of included studies.

**Figure 3:**
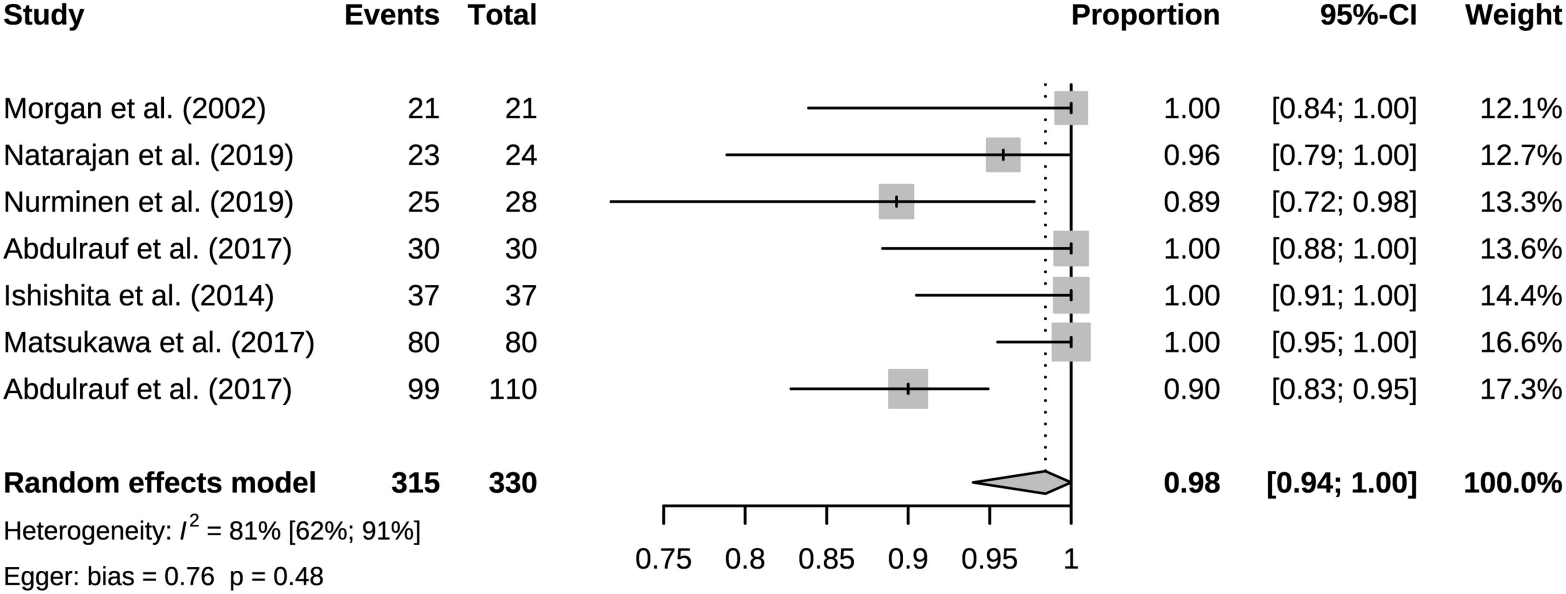
Short-term follow-up bypass patency of included studies.

**Figure 4:**
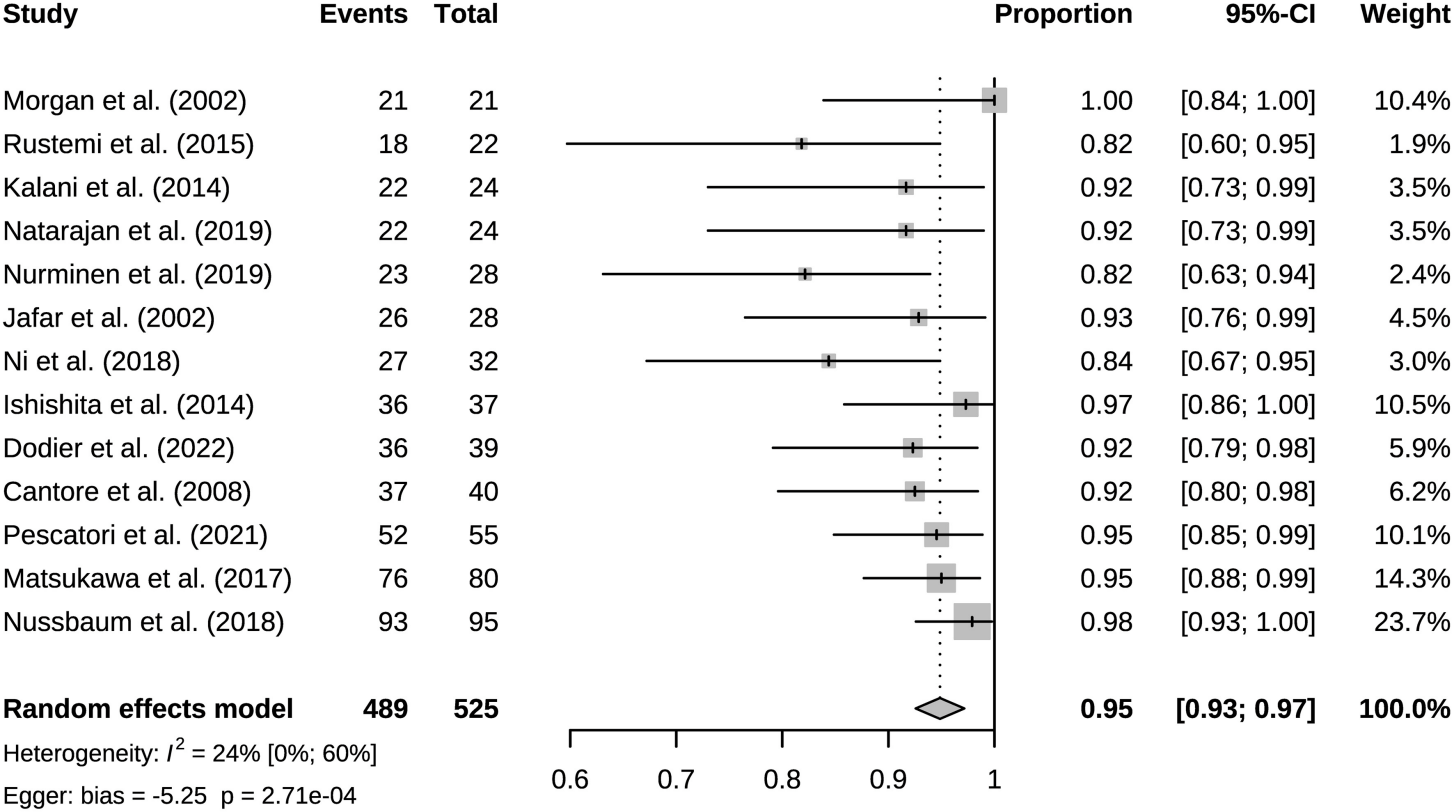
Long-term follow-up bypass patency of included studies.

**Figure 5:**
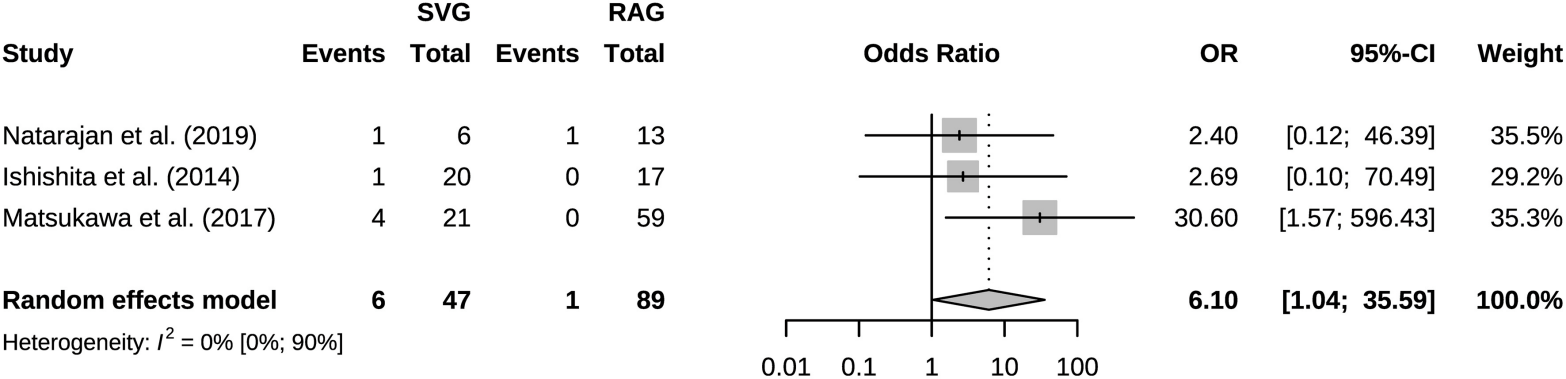
Forest plot showing occlusion rate of saphenous vein graft was higher than radial artery graft.

**Figure 6:**
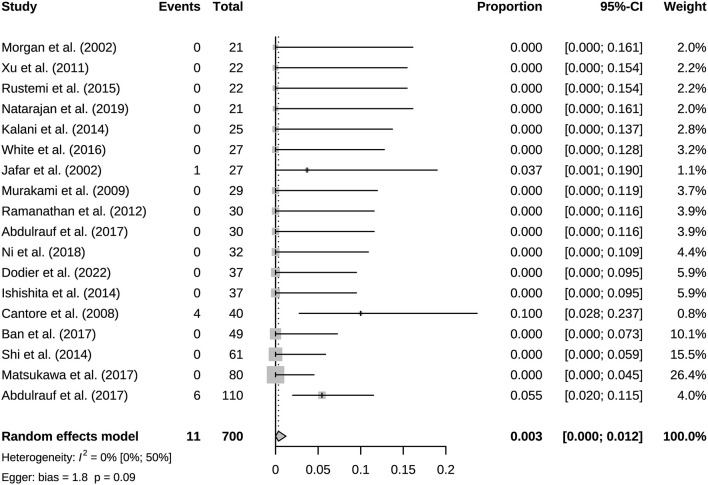
Short-term follow-up surgery-related mortality of included studies.

**Figure 7:**
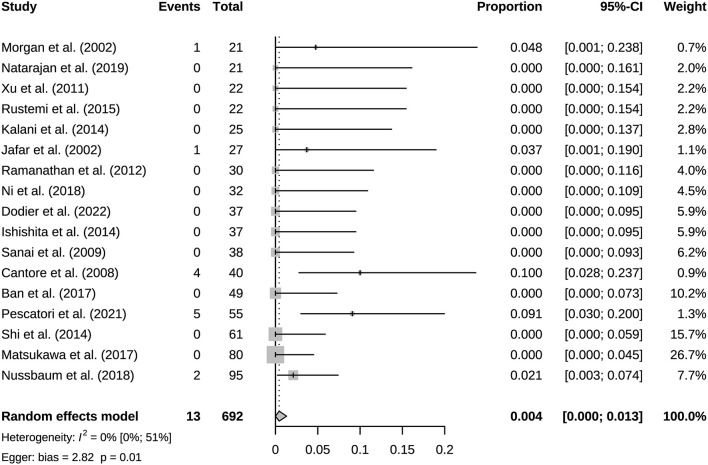
Long-term follow-up surgery-related mortality of included studies.

A total of 941 bypasses were performed across all studies, including 214 (23%) low flow bypasses and 727 (77%) high flow bypasses. The radial artery was used as a graft in 239 bypasses, and the saphenous vein was used as a graft in 228 bypasses. The study of Natarajan et al. included 3 Y-shaped bypasses using the radial and saphenous veins as grafts (Table 2). Both patency and mortality are reported at short-term and long-term follow-up, and patency is also reported postoperatively (Table 3). Postoperative bypass patency was available for a total of 7 studies, and the pooled patency rate was 99% (95% CI 0.98–1.00) (Figure 2). The short-term follow-up patency rate of pooled 7 studies was 98% (95% CI 0.94–1.00) (Figure 3). The heterogeneity was significant, *I*^2^ = 81% (95% CI 62%−91%). A total of 13 studies reported long-time followed up patency, and the pooled patency rate was 95% (95% CI 0.93–0.97) (Figure 4). Four studies compared long-term patency rates for high-flow vs. low-flow bypasses and the result showed no differences between them (OR 1.89 95% CI 0.50–7.15) (Supplementary Figure S1). Saphenous vein grafts (SVG) have higher occlusion rates compared with radial artery grafts (RAG) (OR 6.10 95% CI 1.04–35.59), pooled from 3 studies (Figure 5). The long-term pooled patency rates of high-flow, low-flow, SVG and RAG were 95%, 96%, 93%, and 96% respectively (Supplementary Figures S2–S5). A total of 18 studies were pooled, and 11 people died in the short-term follow-up (*n* = 700), and the pooled mortality rate was 0.3% (95% CI 0.000–0.012) (Table 3, Figure 6). During the long-term follow-up, 13 people died (*n* = 692). The pooled mortality rate was 0.4% (95% CI 0.000–0.013), and 17 studies were pooled (Table 3, Figure 7).

**Table 2:** Types of EC–IC bypass used in the included studies.

**Study**	**LF**	**HF**	**SVG**	**RAG**	**Double STA-MCA**
Morgan et al. (20)	0	21 (100)	21 (100)	0	0
Jafar et al. (21)	0	28 (100)	28 (100)	0	0
Cantore et al. (22)	0	40 (100)	40 (100)	0	0
Sanai et al. (13)	9 (23.68)	29 (76.32)	NR	NR	0
Murakami et al. (23)	17 (58.62)	12 (41.38)	12 (41.38)	0	0
Xu et al. (24)	2 (9.09)	20 (90.91)	20 (90.91)	0	0
Ramanathan et al. (25)	7 (23.33)	23 (76.67)	NR	NR	0
Shi et al. (26)	0	61 (100)	16 (26.23)	45 (73.77)	0
Ishishita et al. (27)	0	37 (100)	20 (54.05)	17 (45.95)	0
Kalani et al. (28)	22 (88)	3 (12)	1 (4)	1 (4)	1 (4)
Rustemi et al. (29)	22 (100)	0	0	0	0
White et al. (30)	9 (33.33)	18 (66.67)	16 (59.26)	0	0
Ban et al. (14)	30 (61.22)	19 (38.78)	14 (28.57)	5 (10.2)	0
Abdulrauf et al. (31)	0	30 (100)	0	30 (100)	0
	0	110 (100)	NR	NR	0
Matsukawa et al. (32)	0	80 (100)	21 (26.25)	59 (73.75)	0
Nussbaum et al. (33)	68 (71.58)	27 (28.42)	NR	NR	0
Ni et al. (34)	0	32 (100)	0	32 (100)	0
Nurminen et al. (35)	12 (42.86)	16 (57.14)	12 (42.86)	1 (3.57)	3 (10.71)
Natarajan et al. (36)	8 (33.33)	16 (66.67)	6 (25) (include 3 Y bypasses)	13 (54.17) (include 3 Y bypasses)	0
Pescatori et al. (37)	0	55 (100)	6 (10.91)	49 (89.09)	0
Dodier et al. (38)	7 (17.5)	33 (82.5)	0	0	33 (82.5)

Data presented as n (%). LF, low flow; HF, high flow; SVG, saphenous vein graft; RAG, radial artery graft; STA, superficial temporal artery; MCA, middle cerebral artery; NR, non-reported.

**Table 3:** Primary outcomes of included studies.

**Study**	**Postoperative patency rate**	**Short-term follow-up patency**	**Long-term follow-up patency**	**Short-term follow-up mortality**	**Long-term follow-up mortality**
Morgan et al. (20)	21 (100)	21 (100)	21 (100)	0	1 (4.76)
Jafar et al. (21)	NR	NR	26 (92.86)	1 (3.7)	1 (3.7)
Cantore et al. (22)	NR	NR	37 (92.5)	4 (10)	4 (10)
Sanai et al. (13)	NR	NR	NR	0	0
Murakami et al. (23)	NR	NR	NR	0	NR
Xu et al. (24)	NR	NR	NR	0	0
Ramanathan et al. (25)	NR	NR	NR	0	0
Shi et al. (26)	NR	NR	NR	0	NR
Ishishita et al. (27)	36 (97.3)	37 (100)	36 (97.3)	0	0
Kalani et al. (28)	NR	NR	22 (91.67)	0	0
Rustemi et al. (29)	22 (100)	NR	18 (81.82)	0	0
White et al. (30)	NR	NR	NR	0	NR
Ban et al. (14)	NR	NR	NR	0	0
Abdulrauf et al. (31)	30 (100)	30 (100)	NR	0	NR
	NR	99 (90)	NR	6 (5.45)	NR
Matsukawa et al. (32)	80 (100)	80 (100)	76 (95)	0	0
Nussbaum et al. (33)	NR	NR	93 (97.89)	NR	2 (2.11)
Ni et al. (34)	31 (96.88)	NR	27 (84.38)	0	0
Nurminen et al. (35)	NR	25 (89.29)	23 (82.14)	NR	NR
Natarajan et al. (36)	23 (95.83)	23 (95.83)	22 (91.67)	0	0
Pescatori et al. (37)	NR	NR	52 (94.55)	NR	5 (9.09)
Dodier et al. (38)	NR	NR	36 (92.31)	0	0

Data presented as n (%). NR, non-reported.

### 3.3. Neurological function score and complication

**Figure 8:**
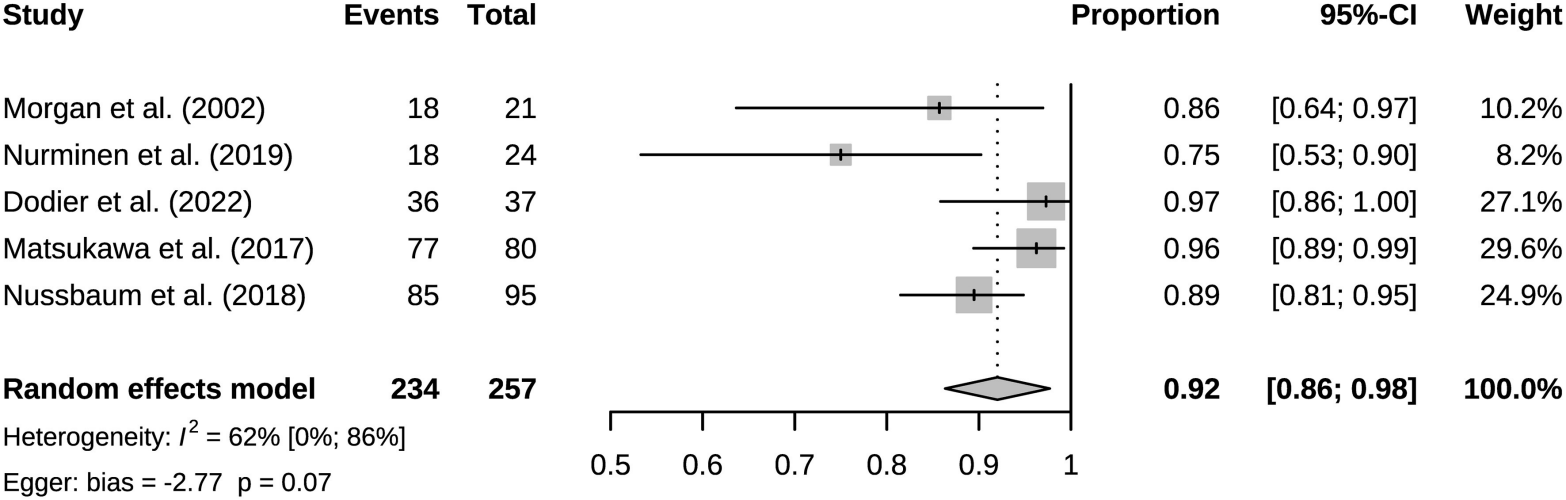
Long-term follow-up proportion of patients with 0–2 points on the modified Rankin scale.

A total of six studies reported mRS scores and two reported GOS scores (Table 4). Compared with preoperatively, the mRS scores of most studies improved significantly, and the number of 0 scorers increased. However, mRS worsening during follow-up was observed in the Nurminen's and Morgan's studies. We pooled the proportion of patients with follow-up mRS 0–2 scores from five studies, 92% (95% CI 0.86–0.98) (Figure 8). Relatively significant heterogeneity was observed, *I*^2^ = 62% (95% CI 0%−86%). Complication rates were available for a total of nine studies (Table 5). The incidence of long-term follow-up complications: ischemic 3% (95% CI 0.01–0.06), hemorrhagic 1% (95% CI 0.00–0.03), neurological deficit 1% (95% CI 0.00–0.03), other complications 3% (95% CI 0.01–0.06) (Supplementary Figures S6–S9).

**Table 4:** Neurological function score of included studies.

**Study reporting mRS**	**Evaluation timing**	**mRS**						
		**0**	**1**	**2**	**3**	**4**	**5**	**6**
Matsukawa et al. (32)	Preoperative	30 (37.5)	32 (40)	15 (18.75)	0	1 (1.25)	2 (2.5)	0
	Discharge	36 (45)	21 (26.25)	17 (21.25)	3 (3.75)	3 (3.75)	0	0
	Follow-up	49 (61.25)	19 (23.75)	9 (11.25)	2 (2.5)	1 (1.25)	0	0
Nurminen et al. (35)	Preoperative	2 (8.33)	15 (62.5)	2 (8.33)	2 (8.33)	1 (4.17)	2 (8.33)	0
	Follow-up	6 (25)	6 (25)	6 (25)	3 (12.5)	2 (8.33)	1 (4.17)	0
Morgan et al. (20)	Preoperative	6 (28.57)	14 (66.67)	1 (4.76)	0	0	0	0
	Follow-up	12 (57.14)	5 (23.81)	1 (4.76)	0	0	1 (4.76)	2 (9.52)
Nussbaum et al. (33)	Follow-up	85 (89.47)			4 (4.21)	2 (2.11)	2 (2.11)	2 (2.11)
**Other mRS formats**								
Dodier et al. (38)	Preoperative	median 2						
	Follow-up	Improve/total 36 (97.3) mRS 0–2 36 (97.3)						
Cantore et al. (22)	Follow-up	Improve/total 35 (87.5)						
**Study reporting GOS**	**Evaluation timing**	**GR**			**MD**		**SD**	
Ishishita et al. (27)	Follow-up	37 (100)			0		0	
Murakami et al. (23)	Follow-up	23 (79.31)			2 (6.9)		4 (13.79)	

Data presented as n (%). mRS, modified Rankin scale; GOS, glasgow outcome scale; GR, good recovery; MD, moderately disabled; SD, severely disabled; NR, non-reported.

**Table 5:** Secondary outcomes of included studies.

**Study**	**Ischemic**	**Hemorrhagic**	**Neurological deficits**	**Other**
Morgan et al. (20)	0	0	0	0
Jafar et al. (21)	1 (3.7)	NR	1 (3.7)	NR
Xu et al. (24)	1 (4.55)	0	0	0
Ishishita et al. (27)	1 (2.7)	0	0	2 (5.41)
Kalani et al. (28)	0	1 (4)	1 (4)	1 (4)
Rustemi et al. (29)	1 (4.55)	0	0	0
Nussbaum et al. (33)	6 (6.32)	7 (7.37)	4 (4.21)	7 (7.37)
Natarajan et al. (36)	3 (14.29)	NR	NR	NR
Dodier et al. (38)	1 (2.7)	0	0	4 (10.81)

Data presented as n (%). NR, non-reported.

## 4. Discussion

This study included 21 eligible studies, involving a total of 915 patients. Bypass patency was high postoperatively and during follow-up. Bypass patency rate of post-operation, the short-term follow-up, and long-term follow-up were 99%, 98%, and 95%, respectively. More than three quarters of bypasses are high flow bypasses. HF bypass surgery may have lower patency rates than LF bypass, but comparison based on four studies did not show meaningful results. Our results basically consistent with a previous study of patency rates in 430 bypass surgeries (39). Its aneurysm group had an overall patency rate of 95%. Its overall patency was lower for HF bypasses than for LF bypasses, however there was no difference in long-term follow-up. With the grafts involved, it appears that the HF is prone to result in occlusion. For instance, the vasospasms of graft, vascular intimal injuries and mismatch of arteries caliber could lead to the formation of thrombus and grafts occlusion. Generally, the LF bypass is recommended due to its safety (40). Under particular circumstances, the combination of blood flow assessment is needed when applying the HF bypass to maximize the safety (12). SVG have higher occlusion rates than RAG. But only three studies were compared, and its extrapolation is limited. SVG and RAG are the most used grafts in bypass surgery. They have different characteristics, for example the radial artery has good thickness and arterial endothelium, but the saphenous vein can provide higher flow. Predominance of the radial artery in the coronary arteries has been established, however more research is needed on cerebral revascularization (41). Based on the existing evidence, we recommend that the radial artery has a greater advantage when the flow rate can be met, which is in line with the current views of most researchers (42). Mortality associated with bypass surgery in this study was extremely low, reflecting its robust safety. And the vast majority of patients showed good prognosis (mRS 0–2) after surgery. The postoperative mRS score is affected by the preoperative status. Considering some patients with poor preoperative scores, the actual improvement in prognosis should be slightly better than the current results. The risk of postoperative complications was low, and they were mostly ischemic.

The results of heterogeneity analysis showed significant heterogeneity in short-term follow-up patency rate and mRS score. This may be influenced in part by the different preoperative status of the patients, such as study by Nurminen et al. (35). The preoperative mRS 3–5 patients were 20.83%, and the postoperative mRS 3–5 patients were 25%. Its preoperative mRS score was the worst of all studies reporting mRS and may have partially influenced the results. Publication bias existed in most studies, except for bypass patency and mortality in long-term follow-up. Sources of publication bias explained by non-comparative studies and small literature numbers, which had less significance for the results.

The quantity and quality of the existing evidence for EC–IC bypass are unsatisfactory. A systematic review that included 20 studies in 2008 showed that EC–IC bypass surgery reduces ischemic stroke risk following therapeutic permanent ICA occlusion for the IAs in anterior circulation (43). This provides guidance for the selection of EC-IC bypass. But considering the sample size and quality of the included studies, the stability of the results is limited. A 2021 systematic review examined the role of EC–IC bypass in the treatment of blood-vesting aneurysms of the ICA (44). However, the sample sizes of the included studies were all <20, and there is no prospective study, precluding any reliable conclusions. The studies we included contain 4 prospective research, with all sample size more than 20, and overall quality of moderate to high. Consequently, our systematic analysis provides more solid proof of the safety in EC–IC.

Our study still has limitations. Most of the studies were retrospective, although the average quality of the studies was moderate to high. In recent years, the development of interventional therapy, especially FD, has greatly reduced the application of bypass surgery for the treatment of giant and complex IA, which poses a challenge for prospective studies of bypass surgery. There is a lack of sufficient studies reporting comparisons of preoperative status to assess postoperative improvement. In addition, subgroup information such as aneurysm rupture and balloon occlusion test (BOT) information is lacking.

In summary, current evidence suggests a high safety profile of EC–IC bypass therapy for IA in anterior circulation. But the level of evidence is low. In the era of endovascular treatment of IA, there are still complex aneurysms that are not suitable for simple endovascular treatment and microsurgical clipping. The combination of EC–IC bypass and other surgical methods such as parent artery occlusion still has an irreplaceable role. Its safety remains to be determined by evidence from large randomized controlled trials (RCT) and high-quality meta-analyses based on RCTs.

## Data availability statement

The original contributions presented in the study are included in the article/Supplementary material, further inquiries can be directed to the corresponding author.

## Author contributions

YC, PC, GD, and GG performed literature search, data extraction, and statistical analysis. RL and ZL completed the visualization. YC drafted the manuscript. YC and GG revised the manuscript. GG provided funding. All authors contributed to the study design conception. All authors contributed to the article and approved the submitted version.
