# Supplementary material for: Extracranial-intracranial bypass surgery for intracranial aneurysm of the anterior cerebral circulation: A systematic review and meta-analysis
Source: Front Neurol. 2023 Mar 31;14:1174088. doi: 10.3389/fneur.2023.1174088 (PMC10102499; doi:10.3389/fneur.2023.1174088)
Supplement: Supplementary file 1 [file Data_Sheet_1.docx]

Supplementary Material

Extracranial-intracranial bypass surgery for intracranial aneurysm of the anterior cerebral circulation: a systematic review and meta-analysis

Yang Chen^†^, Pengyu Chen^†^, Guosheng Duan^†^, Ren Li, Ziao Li and Geng Guo^*^

†These authors made the same contribution to this work

*** Correspondence:** Geng Guo: [guogeng973@163.com](mailto:guogeng973@163.com)

# Supplementary Figures


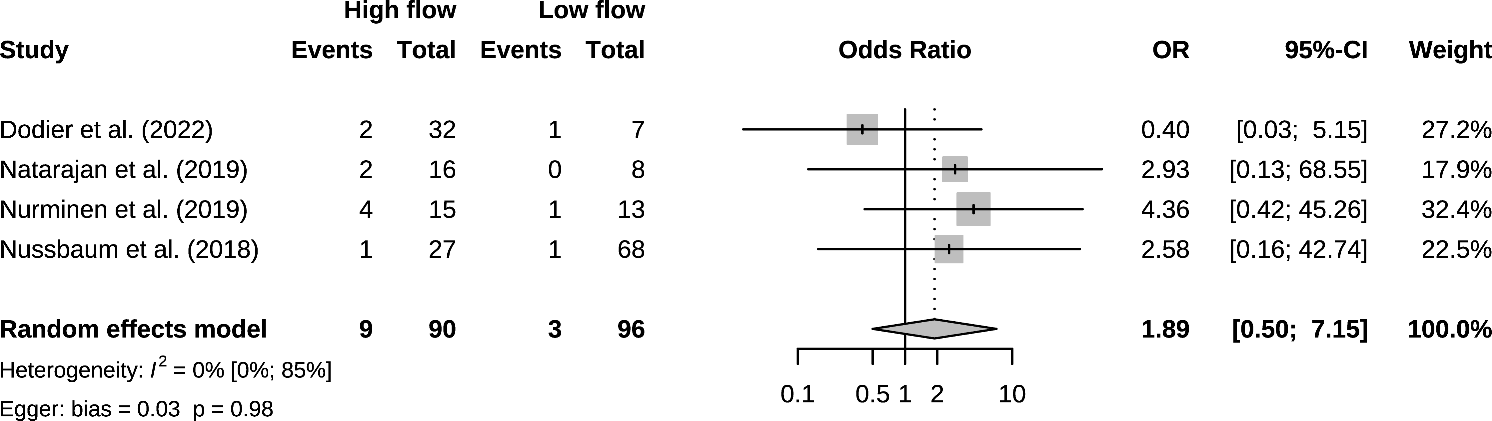


**Supplementary Figure 1.** Forest plot showing bypass patency of high flow and low flow were no difference.


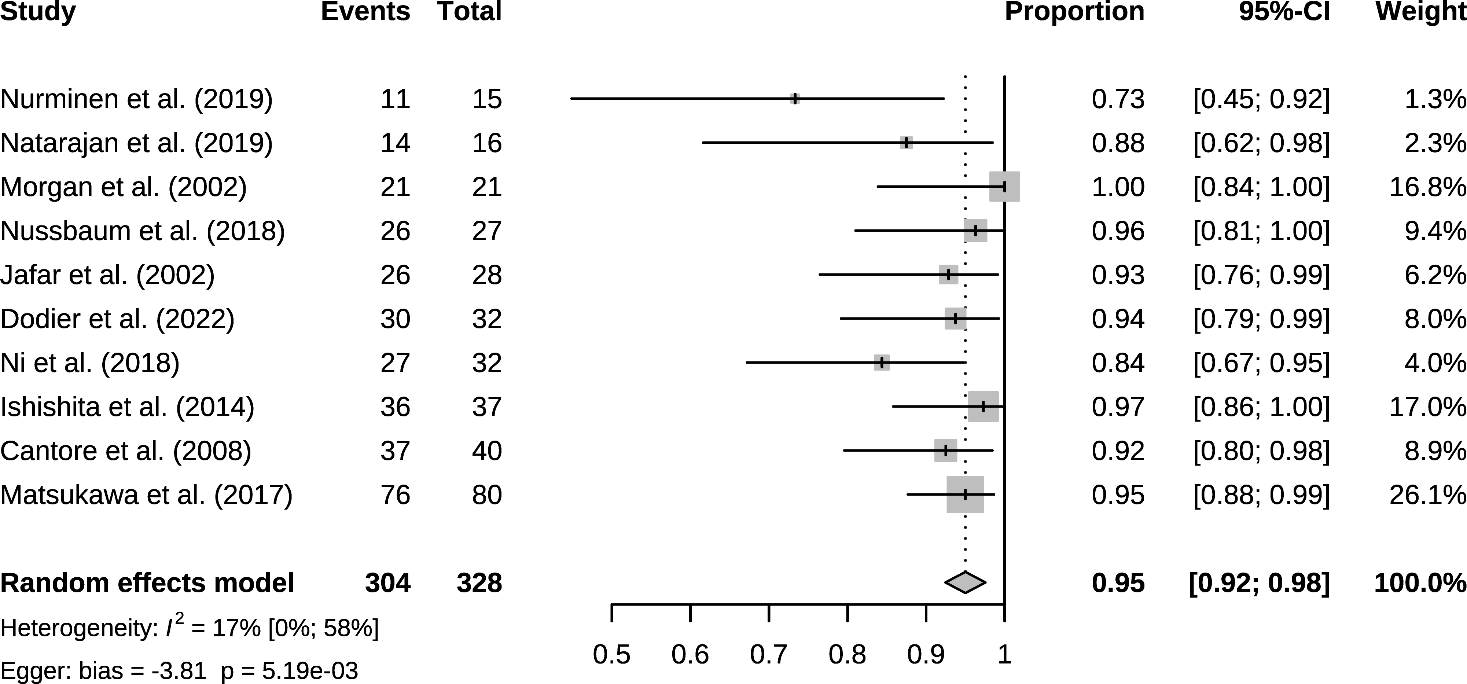


**Supplementary Figure 2.** Patency of high flow bypass during long-term follow-up.


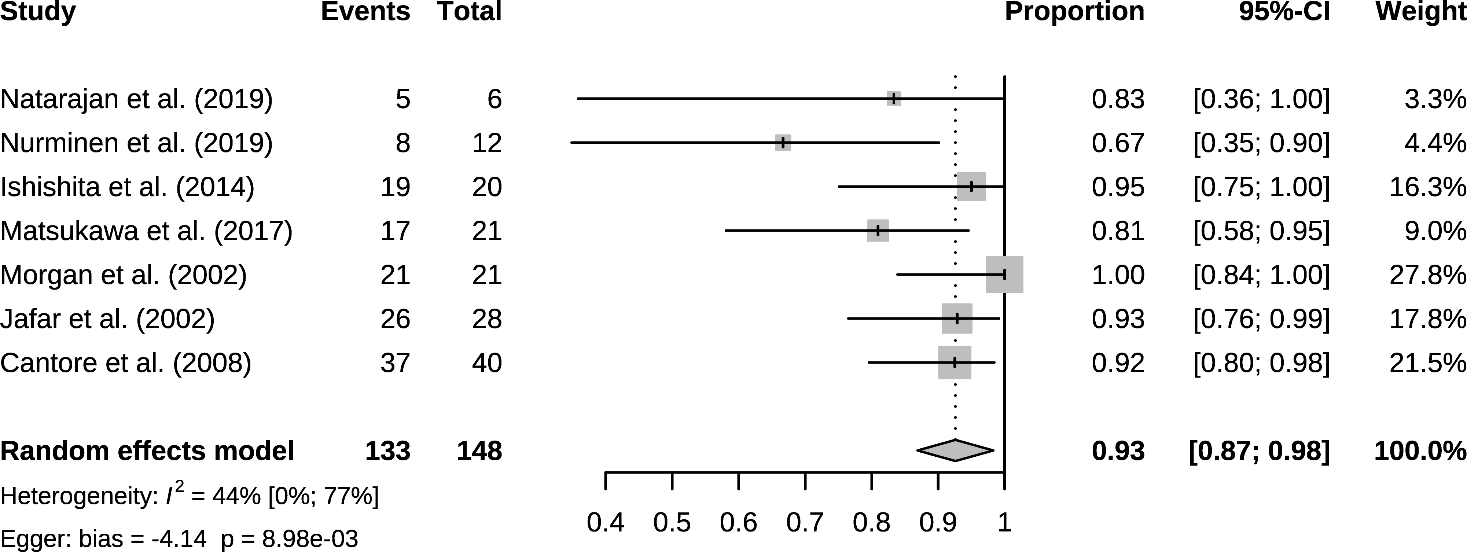


**Supplementary Figure 3.** Patency of low flow bypass during long-term follow-up.


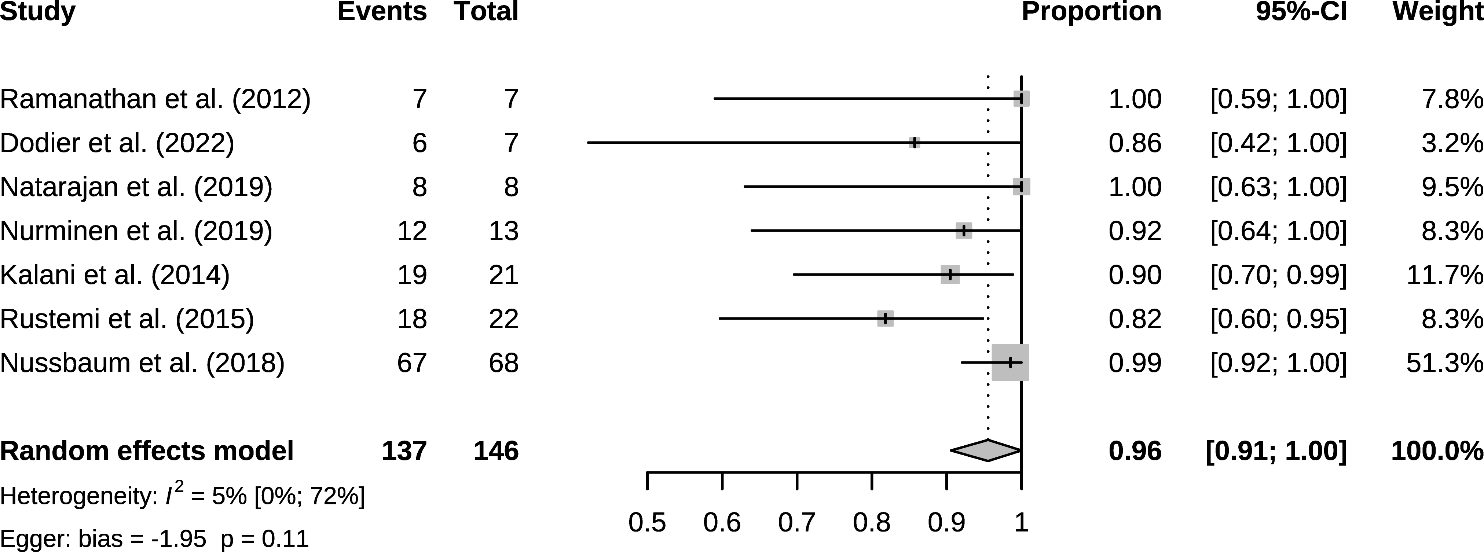


**Supplementary Figure 4.** Patency of saphenous vein graft during long-term follow-up.


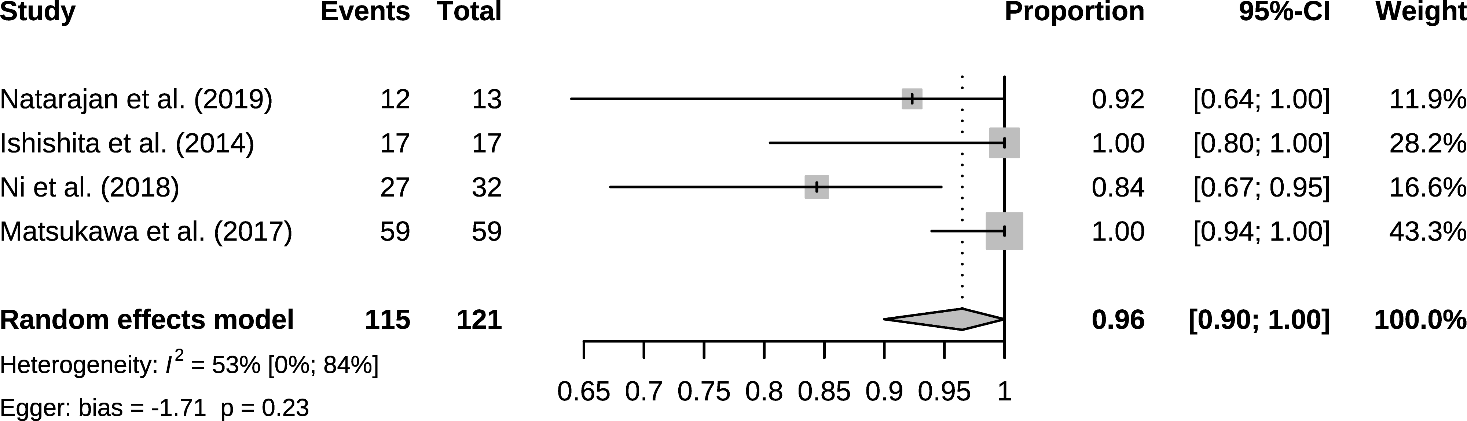


**Supplementary Figure 5.** Patency of radial artery graft during long-term follow-up.


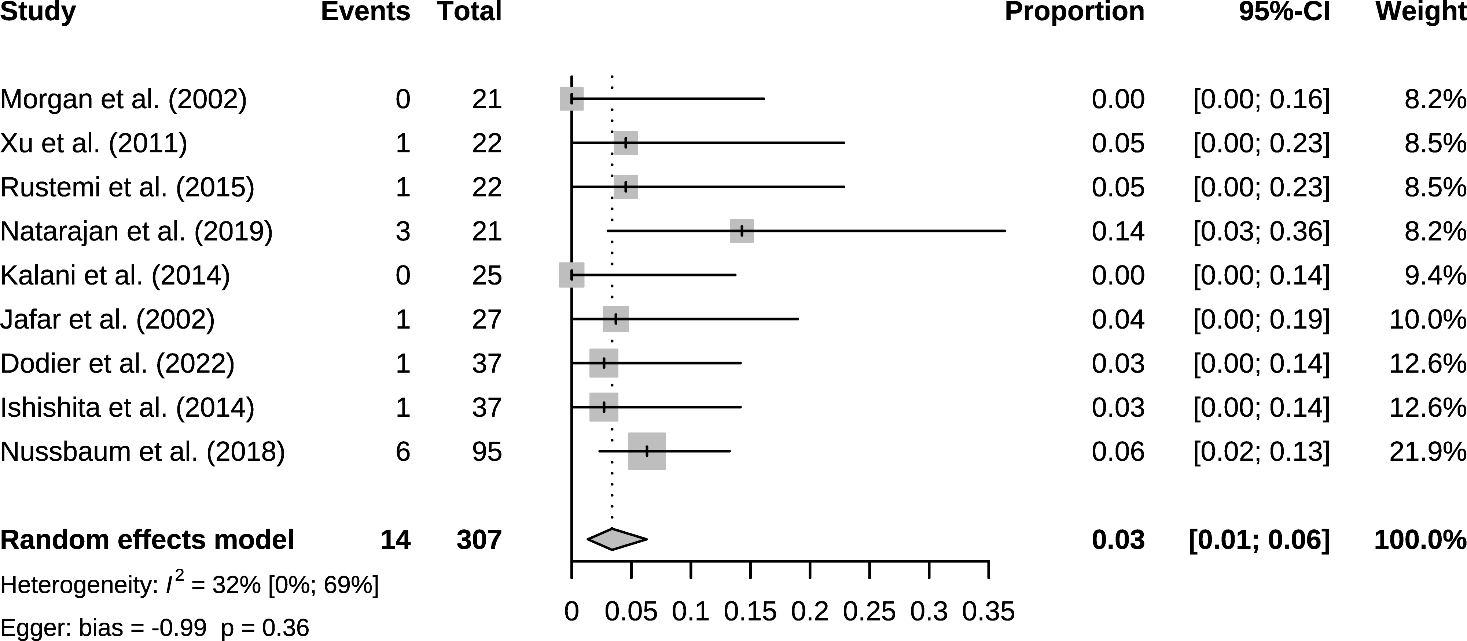


**Supplementary Figure 6.** Incidence rate of ischemic complications during long-term follow-up.


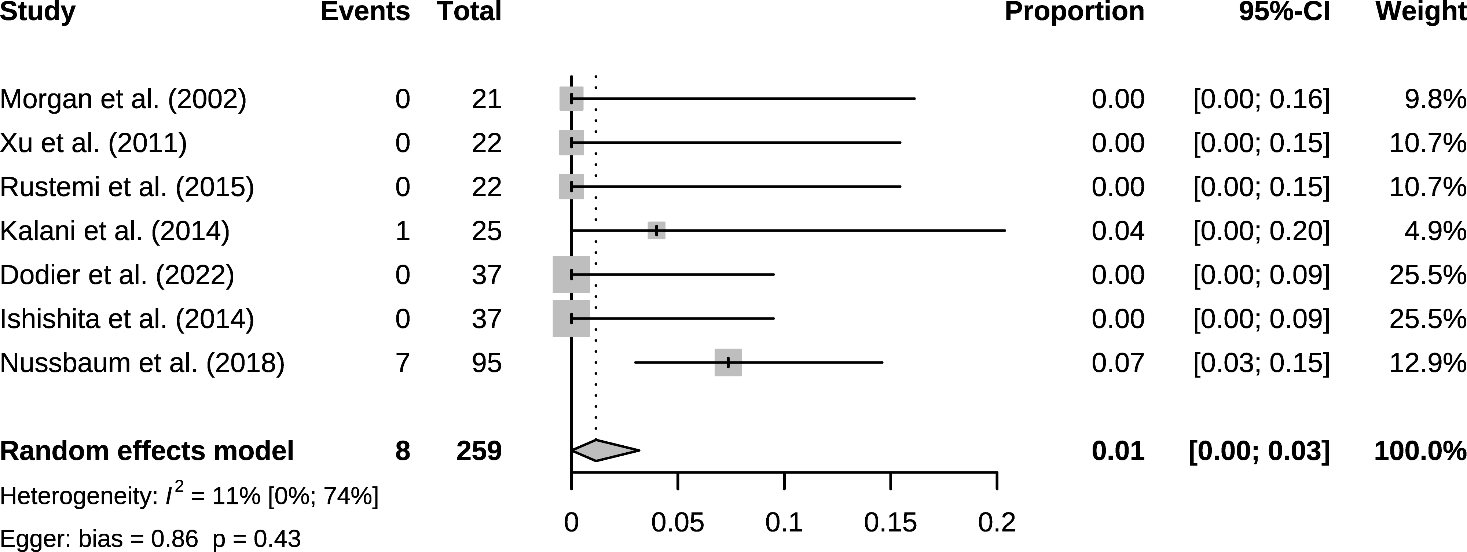


**Supplementary Figure 7.** Incidence rate of hemorrhagic complications during long-term follow-up.


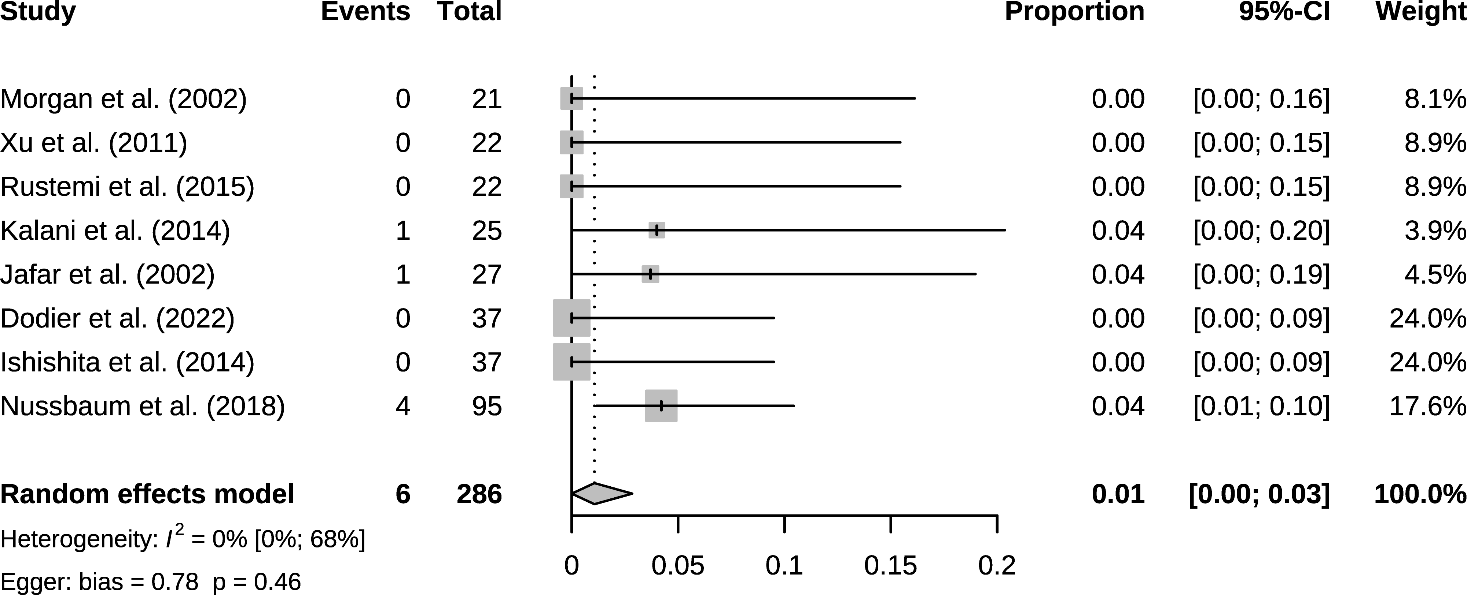


**Supplementary Figure 8.** Incidence rate of neurological deficit complications during long-term follow-up.


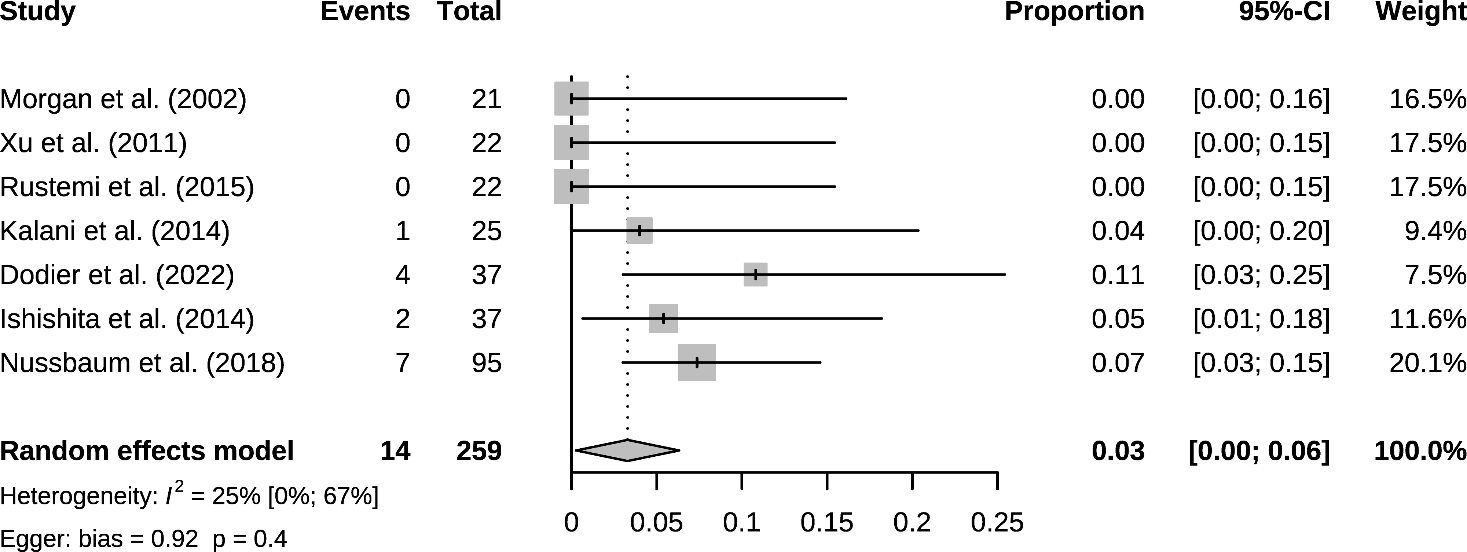


**Supplementary Figure 9.** Incidence rate of other complications during long-term follow-up.
